# Effects of IFN-γ on the immunological microenvironment and TAM polarity in stage IA non-small cell lung cancer and its mechanisms

**DOI:** 10.1186/s12890-023-02809-6

**Published:** 2024-01-22

**Authors:** Weijie Zhao, Huipeng Wang, Xiangwu Zhang, Li Zhang, Wei Pu, Yuhui Ma, Wanling Chen

**Affiliations:** Department of Thoracic Surgery, The Third Affiliated Hospital of Kunming Medical University (Tumor Hospital of Yunnan Province), No. 519, Kunzhou Road, Xishan District, Kunming, Yunnan 650118 China

**Keywords:** Non-small cell lung cancer, IFN-γ, IDO1, Immune microenvironment, Macrophage

## Abstract

**Objective:**

To investigate the effect of interferon-γ (IFN-γ) on the immune microenvironment and the polarity of tumor-associated macrophages (TAMs) in stage IA non-small cell lung cancer (NSCLC) and its mechanisms.

**Methods:**

Human non-small cell lung cancer A549 cells were treated with a series of IFN-γ concentrations (0, 50, 100, 150, 200, 250, and 300 ng/mL). Tumor tissues from patients with stage IA NSCLC were cultured using the air–liquid interface culture technique to establish a tumor microenvironment (TME) organ model. The NSCLC model was constructed by subcutaneously embedding small tumor pieces into the back of nonobese diabetic severe combined immune deficiency (NOD SCID) mice. The size and weight of the tumors were recorded, and the tumor volume was calculated. CCK-8 assays were used to investigate cell proliferation, flow cytometry and TUNEL staining were used to evaluate cell apoptosis, colony formation was investigated by cloning experiments, and cell invasion and migration were evaluated by Transwell assays and scratch tests. The expression of apoptosis-related proteins (Bax, Bcl-2 and C-caspase 3), M2 polarization-related markers (CD163, CD206 and IDO1), and marker proteins of cytotoxic T cells and helper T cells (CD8 and CD4) was detected by Western blot. The expression of Ki-67 and IDO1 was detected by immunohistochemistry, and the levels of IL-6, IL-10, IL-13 and TNF-α were measured by ELISA. The expression of CD68 was measured by RT‒qPCR, and the phagocytosis of TAMs was evaluated by a Cell Trace CFSE kit and cell probe staining.

**Results:**

The proliferation activity of A549 cells increased with increasing IFN-γ concentration and peaked when the concentration reached 200 ng/mL, and the proliferation activity of A549 cells was suppressed thereafter. After treatment with 200 ng/mL IFN-γ, the apoptosis rate of cells decreased, the number of cell colonies increased, the invasion and migration of cells were promoted, the expression of Bax and C-caspase 3 was downregulated, and the expression of Bcl-2 was upregulated in cells and the TME model. In the TME model, CD163, CD206, IDO1 and Ki-67 were upregulated, CD8 and CD4 were downregulated, apoptosis was reduced, the levels of IL-6 and TNF-α were decreased, and the levels of IL-10 and IL-13 were increased. IL-4 induced TAMs to express CD163 and CD206, reduced the levels of IL-6 and TNF-α, increased the levels of IL-10 and IL-13, and weakened the phagocytic function of TAMs. IFN-γ treatment further enhanced the effect of IL-4 and enhanced the viability of A549 cells. IDO1 decreased the viability of T cells and NK cells, while suppressing the effect of IFN-γ. In mice, compared with NSCLC mice, the tumor volume and weight of the IFN-γ group were increased, the expression of CD163, CD206, IDO1, Ki-67 and Bcl-2 in tumor tissue was upregulated, the expression of Bax and C-caspase 3 was downregulated, and apoptosis was reduced. The levels of IL-6 and TNF-α were decreased, and the levels of IL-10 and IL-13 were increased in the serum of mice.

**Conclusion:**

In stage IA NSCLC, a low concentration of IFN-γ promotes the polarization of TAMs to the M2 phenotype in the TME model by upregulating the expression of IDO1, promoting the viability of cancer cells, inhibiting the viability of T cells and NK cells, and thus establishing an immune microenvironment conducive to tumor progression.

**Supplementary Information:**

The online version contains supplementary material available at 10.1186/s12890-023-02809-6.

## Introduction

Lung cancer (LC), one of the most commonly diagnosed cancers, poses an increasing burden, and studies have reported that the incidence of LC will continue to increase in most countries through 2035 [1]. Lung cancer is divided into two major categories, namely, non-small cell lung cancer (NSCLC) and small cell lung cancer (SCLC), and NSCLC is the most common type of lung cancer [2]. At present, the best treatment option for NSCLC patients is still surgery, but only a small number of patients are eligible for surgical resection [3]. The biggest challenge of modern oncology is improving the overall survival rate, and the key to achieving long-term survival is preventing tumor recurrence. Studies have found that some patients with stage IA disease develop local recurrence and metastasis even with the standard treatment of lobectomy [4, 5]. The postoperative locoregional recurrence rate of patients with stage IA NSCLC ranges from 5 to 19%, and nearly 27% of patients with stage IA NSCLC eventually die of recurrence and metastasis within 5 years [6, 7]. Therefore, there is an urgent need to study the specific mechanism of tumor progression in patients with stage IA NSCLC and to provide effective targets for the immune targeted therapy of stage IA NSCLC to enhance the treatment efficacy of early NSCLC.

The tumor microenvironment (TME) in solid tumors is a multidimensional system composed of tumor mass and nonmalignant cells, such as fibroblasts, vascular endothelial cells, and innate and adaptive immune cells, whose immune status is closely related to tumor recurrence, metastasis and prognosis [8, 9]. In recent years, tumor immunotherapy has become an effective antitumor strategy that inhibits tumor metastasis by improving systemic immunity, but the biggest obstacle to this treatment is the immunosuppression caused by the TME [10]. Many preclinical studies of lung cancer have focused on the lung TME. It has been confirmed that the immune microenvironment is closely related to the recurrence of lung cancer in early-stage patients [11, 12]. Therefore, the TME is undoubtedly a potential research target for stage IA NSCLC, which is prone to relapse and metastasis. Macrophages located in the TME are called tumor-associated macrophages (TAMs), which are the main infiltrating immune cells in the TME and play an important role in regulating the immune status of the TME [13]. TAMs are divided into M1 type and M2 type. The M1 type has antitumor properties, while the M2 type has tumor-promoting properties, and the functional phenotype is regulated by molecules in the TME [13, 14]. Studies have reported that with the progression of tumors, TAMs are transformed into the M2 type under the action of induction factors and various cells in the TME to promote tumor progression [15]. Therefore, this study will explore the factors that induce the TAM-to-M2-type transformation and the changes in various cells in the TME of stage IA NSCLC.

Interferon gamma (IFN-γ) is an inflammatory cytokine that plays a key role in tumor immune surveillance [16]. In innate immunity, IFN-γ production is mainly regulated by natural killer cells (NK cells) and NK T cells, while cytotoxic T cells (CD8 +) and helper T cells (CD4 +) are the major paracrine sources of IFN-γ during adaptive immune responses [17]. It is generally believed that in the early stages of tumor formation, T cells control tumor progression by releasing Th-1 cytokines, including IFN-γ, in response to tumor antigens [18]. Studies have shown that IFN-γ can induce the M1 polarization of TAMs, so it has antitumor properties, but recent studies have found that IFN-γ plays multiple roles in the TME. IFN-γ not only has antitumor properties but also can promote tumor progression. IFN-γ signaling can promote the binding of immunosuppressive molecules on the surface membranes of tumor cells and host myeloid cells and upregulate the expression of immunosuppressive proteins on these cells, thereby establishing an immune microenvironment conducive to tumor progression [19]. It has been reported that the concentration of IFN-γ in the TME determines its function. Tumors treated with low doses of IFN-γ become metastatic, while high doses lead to tumor regression [20]. At present, the role of IFN-γ in tumor progression is still controversial, and its role in stage IA NSCLC is not clear.

Indoleamine 2,3-dioxygenase 1 (IDO1) is a rate-limiting enzyme in the pathway that degrades tryptophan into kynurenines. In the tumor microenvironment, IDO1 is expressed by antigen-presenting cells such as macrophages, dendritic cells and tumor cells. Studies have shown that IFN-γ can induce IDO1 expression in macrophages at the transcriptional level [21, 22]. In addition, coexpression of IDO1 and PD-L1 in lung adenocarcinoma was found to indicate a progressive state of the tumor [23]. This suggests that IDO1 may be an important regulatory protein that causes tumor immunosuppression and plays an important role in tumor progression and recurrence.

The aim of this study was to investigate the regulatory effect of IFN-γ on the M2 polarization of TAMs and the mechanism by which IFN-γ controls the progression of stage IA NSCLC through IDO1 to provide a new target and reference for the treatment of stage IA NSCLC.

## Materials and methods

### Clinical sample collection

Twenty-four patients with stage IA NSCLC aged 18–65 years in the Third Affiliated Hospital of Kunming Medical University (Yunnan Cancer Hospital) from June 2020 to June 2021 were selected, excluding patients with immune system diseases, chronic wasting diseases, infectious diseases and other types of malignant tumors, using Archimedes whole lung real-time navigation electronic bronchoscopy biopsy. The fresh tumor tissues were used to establish an organ model of the tumor immune microenvironment. The study was approved by the ethics committee, and the enrolled patients signed the informed consent form before participating in the study.

### Construction of an organ model of the tumor microenvironment

A tumor microenvironment organ model was constructed based on previous studies [24]. Tumor tissues from patients with stage IA NSCLC were washed twice with DMEM/F12 containing 1 × Normorcin, resuspended in type I collagen gel and placed on precoagulated collagen gel. The gel containing the tissue was then cultured in 1 mL of DMEM/F12 medium containing 50% Wnt3a, RSPO1, and other factors.

### Cell culture

The human non-small cell lung cancer cell line A549 and the human monocytic leukemia cell line THP-1 were purchased from Shenzhen Otwo Biotechnology Co., Ltd. All cells were cultured in RPMI 1640 medium (Gibco, CA, USA) containing 10% fetal bovine serum and 100 U/mL penicillin and streptomycin in a 5% CO_2_ incubator at 37 °C. When the cell confluence reached 80%, the subsequent experiments were carried out. THP-1 cells were induced with 25 nM phorbol 12-myristate-13-acetate (PMA) for 48 h to differentiate into macrophages and then treated with 20 ng/mL IL-4 for 72 h to further differentiate into M2 macrophages.

### Cell transfection

Cells in the logarithmic growth phase were washed with PBS 3 times, digested with 0.25% trypsin solution, seeded in a 24-well plate, and cultured at 37 °C in a 5% CO_2_ incubator for 24 h. si-NC and si-IDO1 were transfected into cells with Lipofectamine® 3000 reagent (Invitrogen, CA, USA) according to the manufacturer's instructions. The transfection efficiency was verified by Western blot, and successfully transfected cells were used for subsequent experiments.

### Isolation and purification of lymphocytes

T cells and NK cells were isolated using a magnetic bead sorting kit (Miltenyi Biotec B. V. & Co. KG, Bergisch Gladbach, Germany). Human peripheral blood was collected, and lymphocytes were isolated. After washing and purification, according to the manufacturer's instructions, the corresponding magnetic beads were added for resuspension, and T cells and NK cells were isolated by chromatography for subsequent experiments.

### Laboratory animals

In this study, nonobese diabetic severe combined immune deficiency (NOD SCID) mice were purchased from the Animal Experimental Center of Kunming Medical University. The mice underwent an adaptation period of one week in SPF-grade cages with a temperature of 22–26 ℃, relative humidity of 52–58%, and light–dark cycle of 12 h/12 h. The NSCLC model was constructed based on previous studies [25]. Fresh tumor tissue samples from patients with stage IA NSCLC were cut into small pieces (2 mm), mixed with 10% Matrigel, and subcutaneously injected into the back of NOD SCID mice. When the subcutaneous tumor of the mice grew to approximately 1000 mm^3^, the transplantation was successful, and the primary generation (F1 generation) subcutaneous transplantation mouse model was established. Then, the subcutaneous tumor was harvested and transplanted into NOD SCID mice according to the above inoculation steps to establish the transplanted mouse model second generation (F2 generation). Then, the NOD SCID mice were bred for 3 generations, and the final animal model was established. The F4 generation NOD SCID mice were randomly divided into 2 groups (5/group): the NSCLC group and the IFN-γ group (200 μg/mL IFN-γ protein was injected into the tail vein). The tumor volume of the mice was measured on Day 10, Day 20 and Day 30. After 30 days, the mice were killed for cervical vertebra dislocation, and the transplanted tumor tissues were collected for follow-up experiments.

### Cell viability test

Cells were seeded into 96-well plates at a density of 1 × 10^5^ cells/well and cultured in a 5% CO_2_ incubator at 37 ℃ for 24 h. CCK-8 reagent was added (10 μL/well), and the culture was continued for 2 h. The 96-well plate was then placed on a microplate reader, and the absorbance value was read at 450 nm.

### Cloning experiment

For this experiment, 1.2% and 0.7% agarose gel were prepared and the 1.2% solution was mixed with culture medium at a ratio of 1:1. The mixture was added to a 6-well plate (1.5 mL per well) and solidified at room temperature. The cells were washed with PBS, digested with trypsin, centrifuged, and resuspended in new culture medium. The cell concentration was adjusted to 1 × 10^5^/mL. Then, the upper layer of gel was added. The 0.7% agarose gel solution was mixed with the culture medium at a ratio of 1:1. Then, 100 μL of cell suspension was added. The solution was mixed well and added to the plate (1.5 mL/well). Cells were cultured in a 5% CO_2_ incubator at 37 ℃ for 10 min. Cells were stained with crystal violet, washed and counted.

### Scratch test

A total of 1 × 10^5^ cells were seeded into a 6-well plate, and when the cells reached 100% confluence, the cells were scratched with a pipette tip. The cells were washed three times with PBS to remove the cell debris, and serum-free medium was added. The cells were incubated at 37 ℃ in a 5% CO_2_ incubator and photographed by a microscope at 0 h and 24 h for analysis.

### Transwell experiment

The matrix gel was added to the upper chamber of the Transwell insert, and 1 × 10^5^ cells were seeded. The culture medium was added to the lower chamber, and after incubation at 37 °C and 5% CO_2_ for 48 h, the Transwell chamber was removed and washed with PBS after removing the medium. Cells were then fixed with methanol for 30 min and stained with 0.1% crystal violet for 20 min. Cells in the upper chamber that did not migrate were gently wiped with a cotton swab and counted under a microscope.

### Flow cytometry

Cells were inoculated into 6-well plates at a density of 1 × 10^5^/mL, cultured overnight, digested with trypsin, washed with PBS, resuspended in 300 μL binding solution, incubated with 10 μL Annexin V-FITC at room temperature for 15 min in the dark, and then stained with 5 μL PI solution. After mixing evenly, the cells were incubated in the dark for 5 min, and the percentage of apoptotic cells was detected by flow cytometry.

### TUNEL staining

TUNEL staining was performed according to the instructions of TUNEL kit (BOSTER, Wuhan, China). Mouse tumor tissue was taken for paraffin section. Sections were routinely dewaxed and then subjected to section digestion. The specimens were added with labeling buffer and labelled at 37°C for 2 h. Afterwards, blocking solution was added to block for 30 min at room temperature. Biotinylated anti-digoxigenin antibody was diluted with SABC (biohao, Wuhan, China) diluent, and after DAB (Solarbio, Beijing, China) staining, hematoxylin (Servicebio, Wuhan, China) was lightly counterstained. Dehydrated, transparent, sealed and observed under microscope (Eclipse 80i, Nikon).

### Detection of tumor-associated macrophage (TAMs) phagocytosis

The phagocytosis of TAMs was detected by a Cell Trace CFSE kit (Thermo Fisher, USA). A549 cells were labeled with Cell Trance Yellow reagent according to the manufacturer's instructions, and 1 × 10^5^ TAMs tagged with GFP were inoculated into 6-well plates. The cells were incubated in RPMI complete medium for 2 h and then mixed with labeled A549 cells. The cells were observed and photographed by fluorescence microscopy, and phagocytosis was detected by flow cytometry.

### Phagocytosis of TAMs was observed by cell probe staining

Phagocytosis of TAMs was detected by a Cell-Tracker Green CMFDA tracer probe (green), and the cells were inoculated into 96-well plates at a density of 1 × 10^5^ cells/well and then cultured in a 5% CO_2_ incubator at 37 ℃ for 24 h. The cells were resuspended with preheated Cell-Tracker staining working solution and incubated for 30 min. Then, the working solution was removed and replaced with culture medium, and the cells were cultured at 37 ℃ for 30 min, washed with PBS, observed and photographed under a fluorescence microscope.

### Real-time quantitative PCR (RT‒qPCR)

Total RNA was extracted from cells and tissues using TRIzol reagent (Invitrogen, CA, USA) and reverse-transcribed into single-stranded complementary DNA (cDNA) using a Prime Script™ RT kit with gDNA Eraser (Takara, Dalian, China). RT‒qPCR was performed in triplicate using SYBR Green mix (Life Technologies, CA, USA) following the manufacturer's instructions. Using GAPDH as an internal control, the relative gene expression was calculated and normalized by the 2^−ΔΔCt^ method. The primer sequences are detailed in Table 1.

**Table 1:** Primer sequences

Gene	Primer	Sequence (5 '-3')
CD68	Forward	5′-CTACTGGCAGAGAGCACTGG-3′
	Reverse	5′-CCGCCATGTAGCTCAGGTAG-3′
GAPDH	Forward	5′- GGAGTCCACTGGTGTCTTCA-3′
	Reverse	5′- GGGAACTGAGCAATTGGTGG-3′

### Western blot

The total protein of cells and tissues was extracted, and the protein concentration was determined by the BCA method. The samples were separated by 10% SDS‒PAGE, transferred to PVDF membranes, immersed in 5% skim milk powder, and blocked at room temperature for 2 h. Prediluted primary antibodies IDO1 (1:1000, Abcam, UK), CD163 (1:1000, Abcam, UK), CD206 (1:1000, Thermo Fisher, USA), CD4 (1:1000, Abcam, UK), CD8 (1:1000, Abcam, UK), Bax (1:1000, Abcam, UK), C-caspase 3 (1:500, Abcam, UK), and Bcl-2 (1:1000, Abcam, UK) were incubated overnight at 4 °C. After the membrane was washed, it was incubated with the corresponding secondary antibody (1:2000, Abcam, UK) at room temperature for 2 h. After ECL visualization, ImageJ was used to analyze the band gray values.

### Enzyme-linked immunosorbent assay (ELISA)

Cellular IL-6, IL-10, IL-13 and TNF-α levels were measured using ELISA kits (Abcam, UK). Cell culture supernatants or mouse serum were collected from each group, and the optical density of each well was determined within 5 min using a microplate reader set at 450 nm according to the manufacturer's instructions. The levels of IL-6, IL-10, IL-13 and TNF-α were calculated according to the standard curve.

### Immunohistochemical staining

The lung tissues of mice were washed with PBS, and paraffin-embedded sections were prepared. After routine dewaxing, the sections were incubated with primary antibodies against IDO1 (1:1000, Abcam, UK) and Ki-67 (1:1000, Abcam, UK) for 30 min at room temperature, washed with PBS, and then treated with secondary antibodies (1:1000, Abcam, UK) at room temperature for 30 min. After removing the excess PBS, the sections were chemically stained, dehydrated and sealed for observation and analysis.

### Statistics and analysis

All experiments in this study were repeated at least 3 times, and the values are expressed as the mean ± standard deviation. GraphPad Prism 7 (GraphPad Software, Inc., San Diego, CA, USA) was used to analyze and plot the data, a t test was used for comparisons between two groups, and one-way ANOVA was used for comparisons among multiple groups. *P* < 0.05 indicates a statistically significant difference.

## Results

### Effects of IFN-γ on the biological behavior of lung cancer cells

**Fig. 1:**
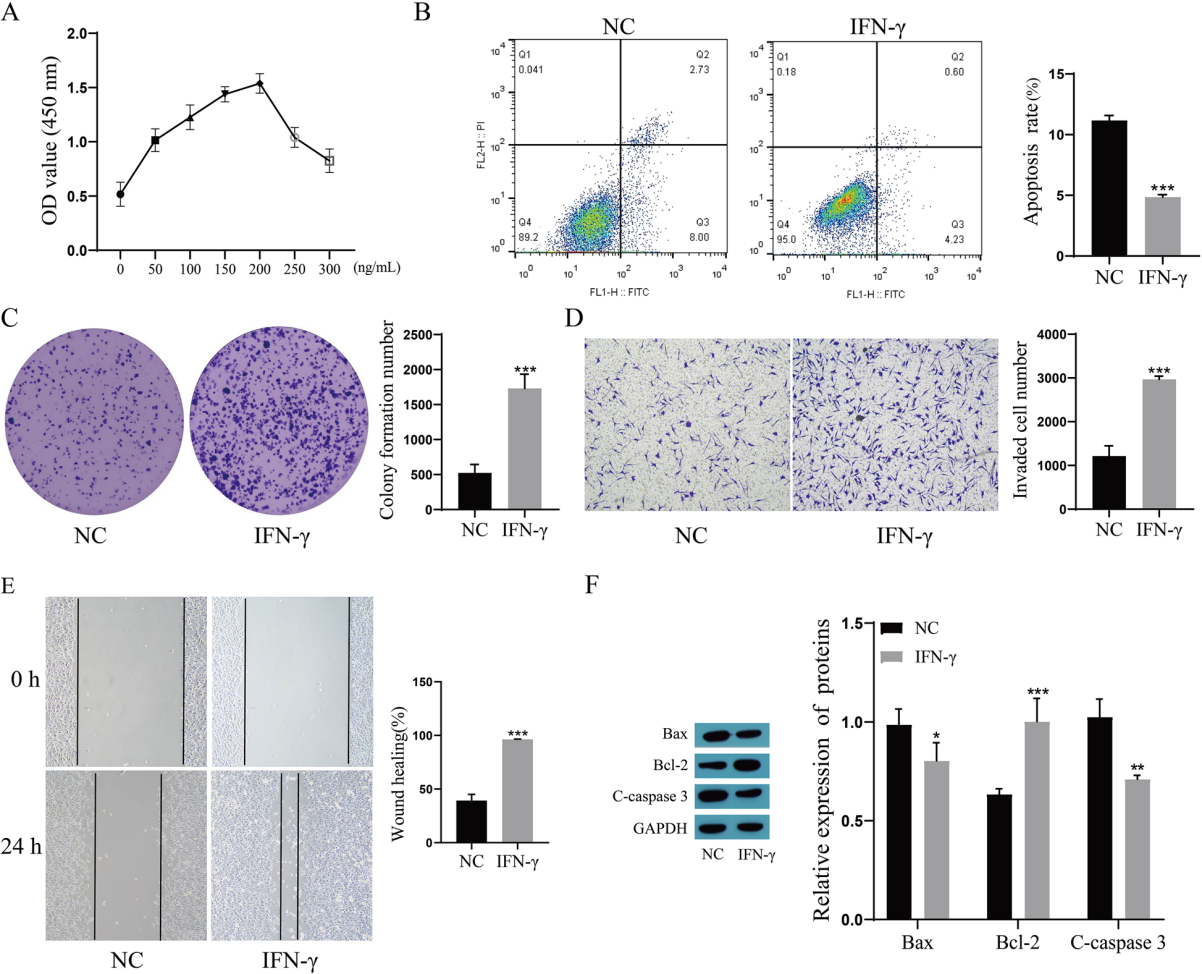
Effects of IFN-γ on the biological behavior of lung cancer cells. **A** Cell proliferation activity was determined by CCK-8; **B** The apoptosis rate was determined by flow cytometry. **C** Cell colony formation was determined by clone assay; **D** Cell invasion ability was determined by Transwell assay; **E** The migration ability of the cells was determined by scratch test. **F** The expression of Bax, Bcl-2 and C-caspase 3 was detected by Western blot. Compared with the NC group, ^*^P < 0.05, ^**^P < 0.01, ^***^P < 0.001

The A549 cells were treated with different concentrations of IFN-γ (0, 50, 100, 150, 200, 250, 300 ng/mL), and the proliferation activity of the cells was detected by a CCK-8 assay. The results showed that the proliferation activity of the cells increased gradually with increasing IFN-γ concentration and peaked at a concentration of 200 ng/mL. After 200 ng/mL, the proliferation activity of the cells gradually decreased (Fig. 1A), so IFN-γ at a concentration of 200 ng/mL was used in the subsequent experiments of this study. The results of apoptosis detection showed that the apoptosis rate of A549 cells treated with 200 ng/mL IFN-γ was significantly reduced compared with that of NC cells (Fig. 1B), and the results of the cloning experiment showed that the colony formation of A549 cells treated with IFN-γ was increased compared with that of NC cells (Fig. 1C). Transwell and scratch assays were used to evaluate the invasion and migration ability of A549 cells. The invasion and migration abilities of cells treated with 200 ng/mL IFN-γ were significantly enhanced compared with those of NC cells (Fig. 1D, E). The expression of apoptosis-related proteins in the cells was examined, and the results showed that the expression of Bax and C-caspase 3 was significantly downregulated and the expression of Bcl-2 was significantly upregulated after treatment with 200 ng/mL IFN-γ (Fig. 1F).

### IFN-γ promotes the M2 polarization of TAMs and progression of stage IA NSCLC

**Fig. 2:**
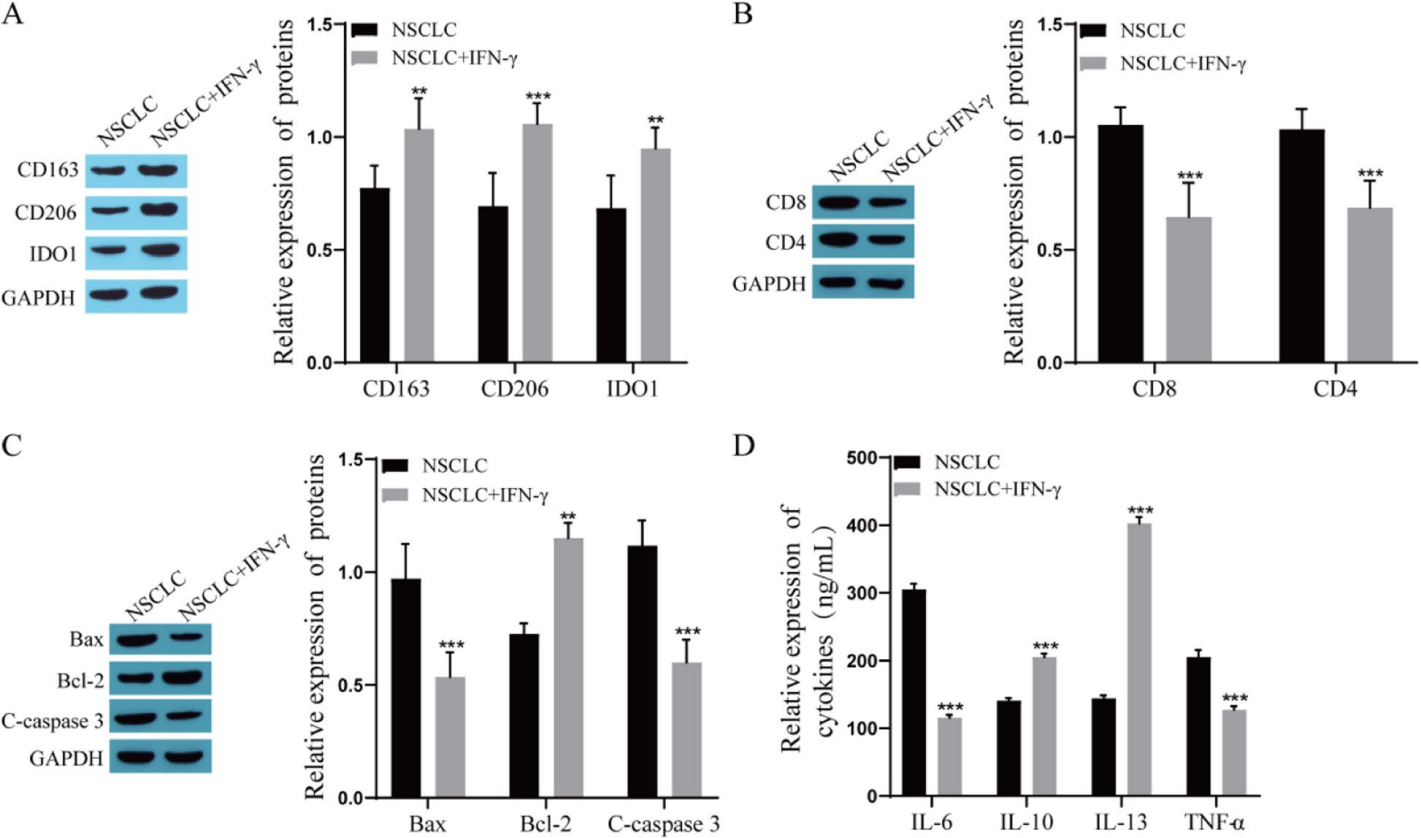
IFN-γ promotes M2 polarization of TAMs and progression of stage IA NSCLC in a TME organ model. **A** The expression levels of CD163, CD206 and IDO1 were detected by Western blot; **B** The expression levels of CD8 and CD4 were detected by Western blot; **C** The expression levels of Bax, Bcl-2 and C-caspase 3 were detected by Western blot; **D** The levels of IL-6, IL-10, IL-13 and TNF-α were measured by ELISA. Compared with the NSCLC group, ^**^ *P* < 0.01, ^***^ *P* < 0.001

To explore the effect of IFN-γ on TAM polarization and tumor cell proliferation in stage IA NSCLC, we established a TME organ model using tumor tissue from stage IA NSCLC patients and treated them with 200 ng/mL IFN-γ. Western blotting was used to detect the expression of CD163, CD206, and IDO1, which are related to the polarization of M2 TAMs. The results showed that the expression of CD163, CD206, and IDO1 was significantly upregulated after IFN-γ treatment (Fig. 2A). We examined the expression of CD8 and CD4 (T-cell-associated proteins) in the TME organ model, and the Western blot results showed that the expression of CD8 and CD4 was significantly downregulated after IFN-γ treatment (Fig. 2B). The expression of apoptosis-related proteins was detected by Western blot, and the results showed that the expression of Bax and C-caspase 3 was significantly downregulated and the expression of Bcl-2 was significantly upregulated after IFN-γ treatment (Fig. 2C). The levels of cytokines were measured by ELISA kits, and the results showed that the levels of IL-6 and TNF-α were significantly decreased and the levels of IL-10 and IL-13 were significantly increased after IFN-γ treatment (Fig. 2D).

### IFN-γ promotes the M2 polarization of macrophages

**Fig. 3:**
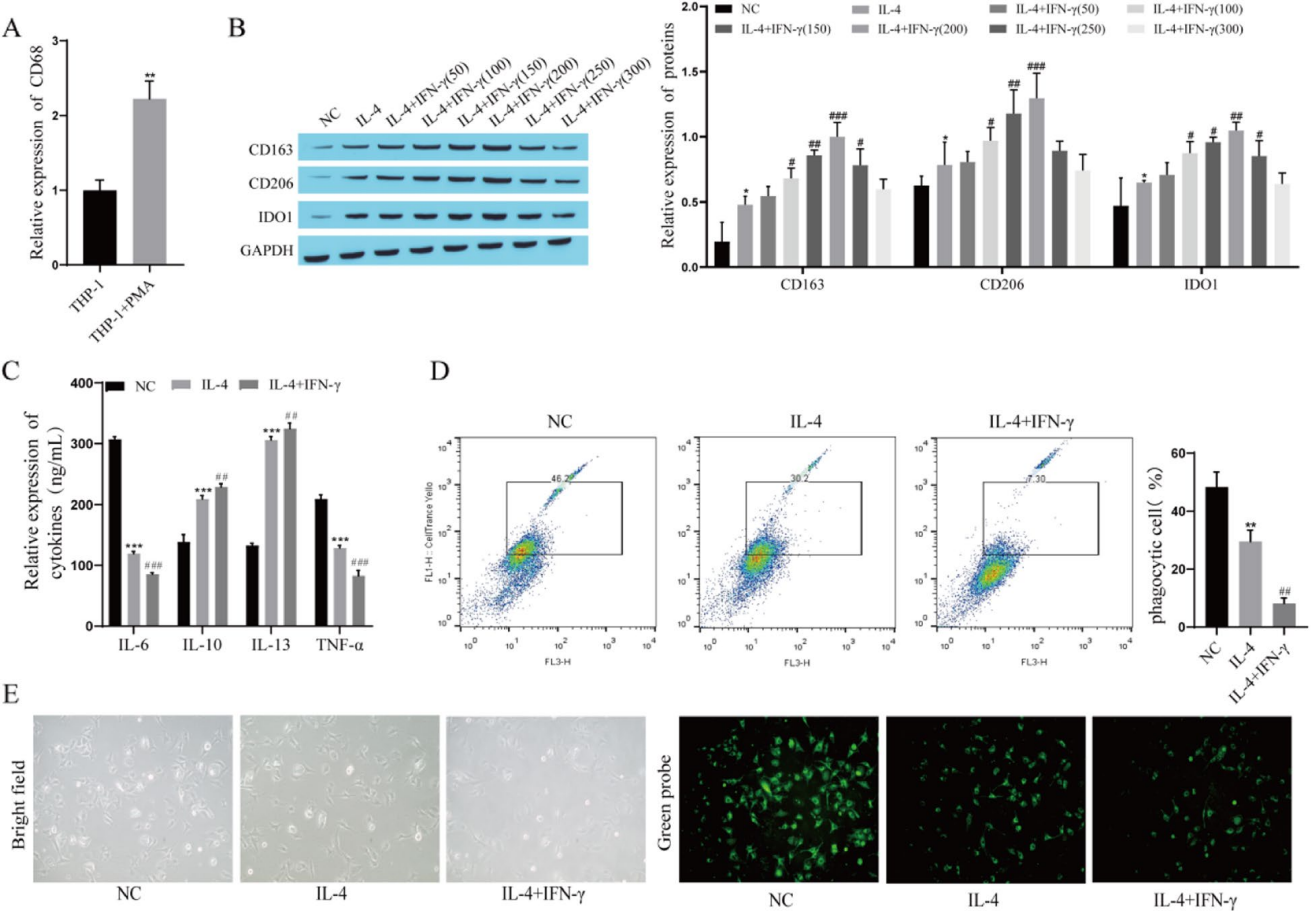
IFN-γ promotes the M2 polarization of macrophages. **A** The expression of CD68 was measured by RT‒qPCR; **B **The expression of CD163, CD206 and IDO1 was detected by Western blot; **C** The levels of IL-6, IL-10, IL-13 and TNF-α were measured by ELISA; **D** The phagocytosis of TAMs was detected by flow cytometry; **E** Phagocytic function of TAMs was observed by cell probe staining. Compared with the THP-1 group or NC group, ^*^P < 0.05, ^**^P < 0.01, ^***^P < 0.001; compared with the IL-4 group,^#^*P* < 0.05, ^##^*P* < 0.01, ^###^*P* < 0.001

To explore the effect of IFN-γ on macrophage polarization, THP-1 cells were first induced to differentiate into CD68 + macrophages by PMA for 48 h, and the expression of CD68 was measured by RT‒qPCR. The results showed that the expression of CD68 was significantly upregulated after PMA induction (Fig. 3A). The results showed that THP-1 cells were successfully differentiated into CD68 + macrophages. CD68 + macrophages were then further differentiated into M2 macrophages using IL-4 treatment and treated with different concentrations of IFN-γ (0, 50, 100, 150, 200, 250, 300 ng/mL) to explore the effect of IFN-γ on macrophage polarization. The Western blot results showed that the expression of the M2 polarization markers CD163 and CD206 was upregulated after IL-4 treatment, and the expression of CD163 and CD206 was further upregulated after IFN-γ treatment in a concentration-dependent manner. The expression of CD163 and CD206 was the highest when the concentration of IFN-γ was 200 ng/mL, while the expression of CD163 and CD206 was downregulated when the concentration of IFN-γ was greater than 200 ng/mL (Fig. 3 B). In subsequent experiments, 200 ng/mL IFN-γ-treated cells were used to observe the changes in other indicators. The ELISA results showed that the levels of IL-6 and TNF-α were significantly decreased and the levels of IL-10 and IL-13 were significantly increased after IL-4 treatment, and IFN-γ treatment further decreased the levels of IL-6 and TNF-α and further increased the levels of IL-10 and IL-13 (Fig. 3C). Subsequently, the phagocytic function of TAMs was examined, and the results of flow cytometry quantification showed that the number of phagocytes was decreased after IL-4 treatment and further decreased after IFN-γ treatment (Fig. 3D). Cells were labeled with a cell probe to observe phagocytosis, which was attenuated after IL-4 treatment and further reduced after IFN-γ treatment (Fig. 3 E).

### Effect of IDO1 on IFN-γ-mediated macrophage M2 polarization

**Fig. 4:**
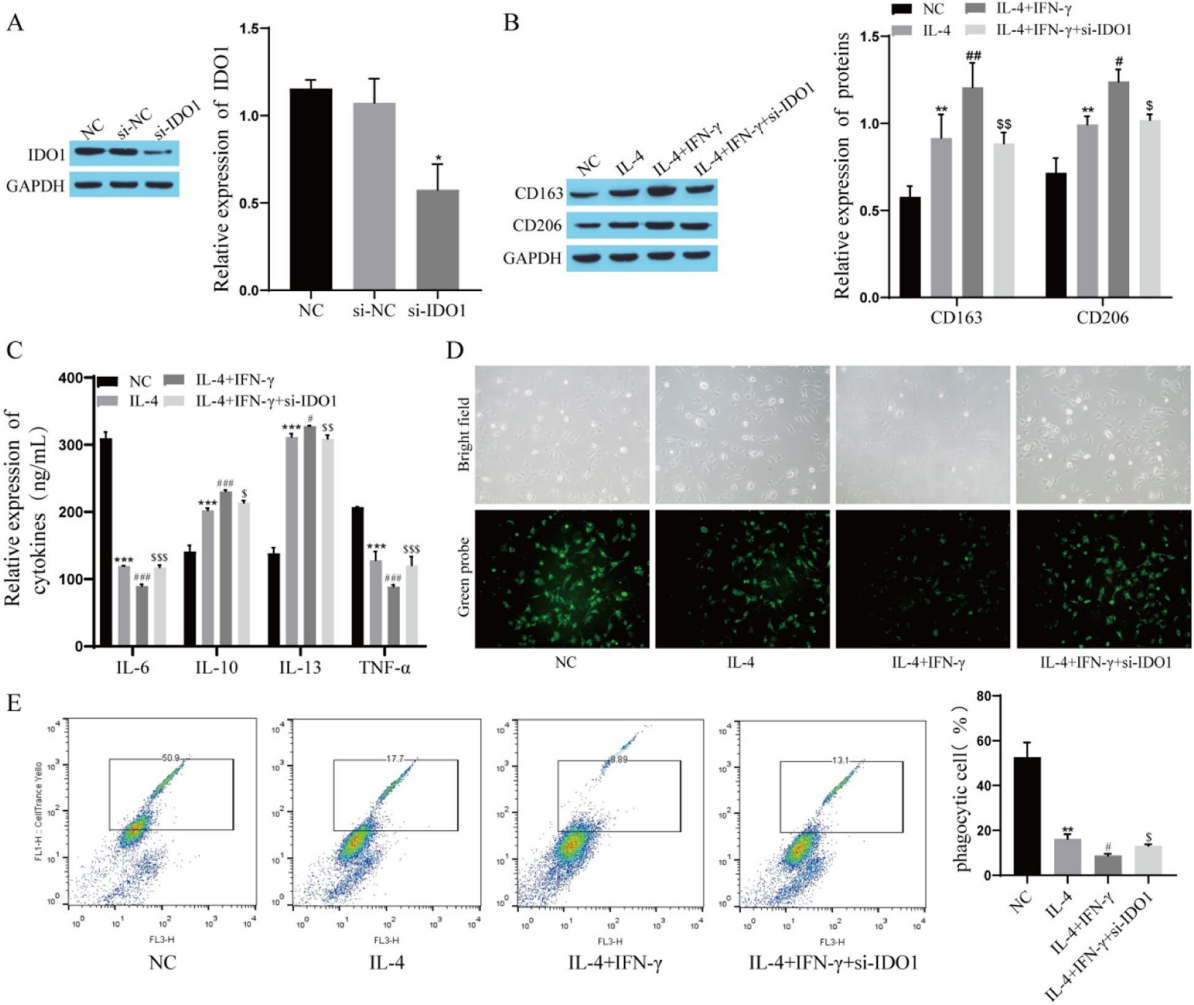
Effect of IDO1 on IFN-γ-mediated macrophage M2 polarization. **A** The transfection efficiency was verified by Western blot; **B** The expression of CD163 and CD206 was detected by Western blot; **C** The levels of IL-6, IL-10, IL-13 and TNF-α were measured by ELISA; **D** The phagocytic function of TAMs was observed by cell probe staining. **E** The phagocytosis of TAMs was detected by flow cytometry. Compared with si-NC group or NC group,^*^ *P* < 0.05,^**^ *P* < 0.01,^***^ *P* < 0.001; Compared with IL-4 group,^#^ *P* < 0.05,^##^ *P* < 0.01,^###^ *P* < 0.001; Compared with IL-4 + IFN-γ group,^$^ *P* < 0.05,^$$^ *P* < 0.01 and ^$$$^ *P* < 0.001

In our study, we found that the expression level of IDO1 increased after IFN-γ treatment in the NSCLC organ model. To explore the effect of IDO1 on the polarization of TAMs, experiments were performed after transfection of si-IDO1 into THP-1 cells. First, Western blot detection of transfection efficiency showed that compared with that in the si-NC group, the expression of IDO1 in the si-IDO1 group was significantly downregulated, indicating successful transfection (Fig. 4A). Western blot results showed that the expression of the M2 polarization markers CD163 and CD206 was upregulated after IL-4 treatment, and the expression of CD163 and CD206 was further upregulated after IFN-γ treatment, while the effect of IFN-γ was attenuated after transfection of si-IDO1 (Fig. 4B). The ELISA results showed that the levels of IL-6 and TNF-α were significantly decreased and the levels of IL-10 and IL-13 were significantly increased after IL-4 treatment. The levels of IL-6 and TNF-α were further decreased, and the levels of IL-10 and IL-13 were further increased after IFN-γ treatment. Transfection of si-IDO1 attenuated the effect of IFN-γ (Fig. 4C). Subsequently, the phagocytosis of TAMs was examined, and the results showed that after IL-4 treatment, the number of phagocytes was reduced and phagocytosis was suppressed. After IFN-γ treatment, the number of phagocytes was further reduced, and the phagocytic effect was further suppressed. After transfection of si-IDO1, the effect of IFN-γ was attenuated (Fig. 4D, E).

### Effects of IFN-γ-treated macrophages on the viability of A549 cells, T cells and NK cells

**Fig. 5:**
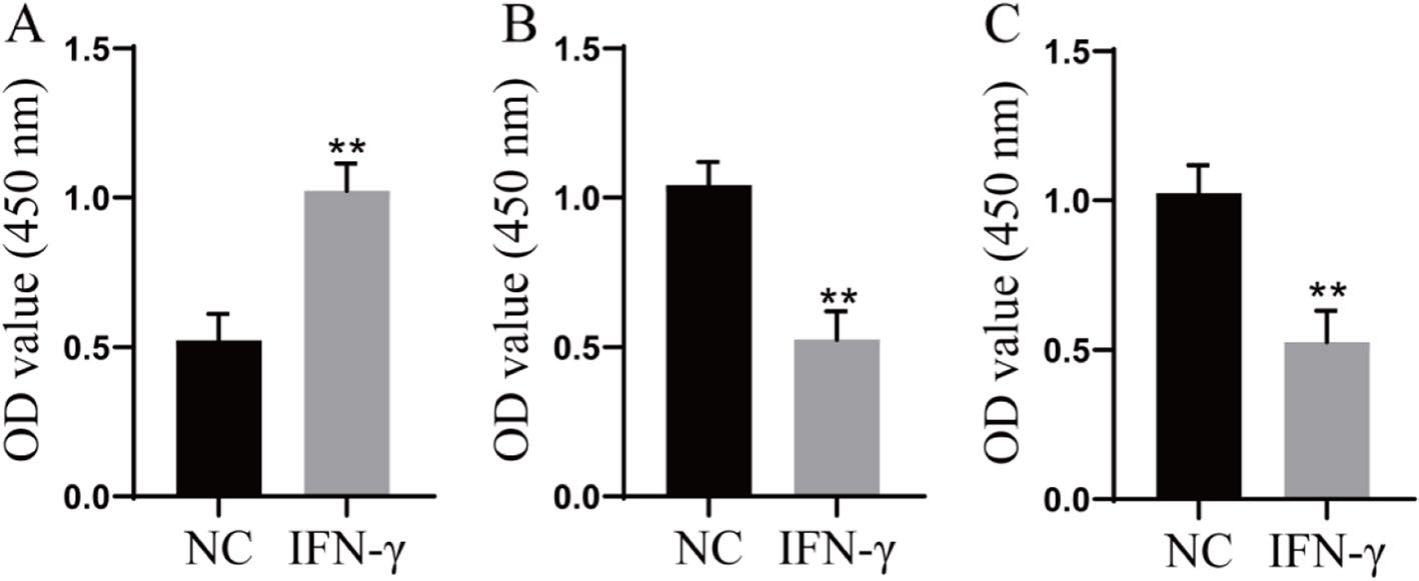
Effects of IFN-γ-treated macrophages on the viability of A549 cells, T cells and NK cells. **A** The viability of A549 cells was determined by a CCK-8 assay; **B** The viability of T cells was determined by a CCK-8 assay; **C** The viability of NK cells was determined by a CCK-8 assay. Compared with the NC group, ^**^ *P* < 0.01

TAMs can interact with other immune cell types in the tumor immune microenvironment. To further explore the effect of IFN-γ on the immune microenvironment, THP-1 cells were induced to differentiate into macrophages by PMA, and the macrophages were treated with 200 ng/mL IFN-γ and cocultured with A549 cells, T cells and NK cells. Cell viability was determined by a CCK-8 assay. The results showed that the viability of A549 cells increased significantly (Fig. 5A), the viability of T cells was significantly decreased (Fig. 5B), and the viability of NK cells was significantly decreased (Fig. 5C) after coculture with IFN-γ-treated macrophages.

### IFN-γ promotes the M2 polarization of TAMs and progression of stage IA NSCLC in mice

**Fig. 6:**
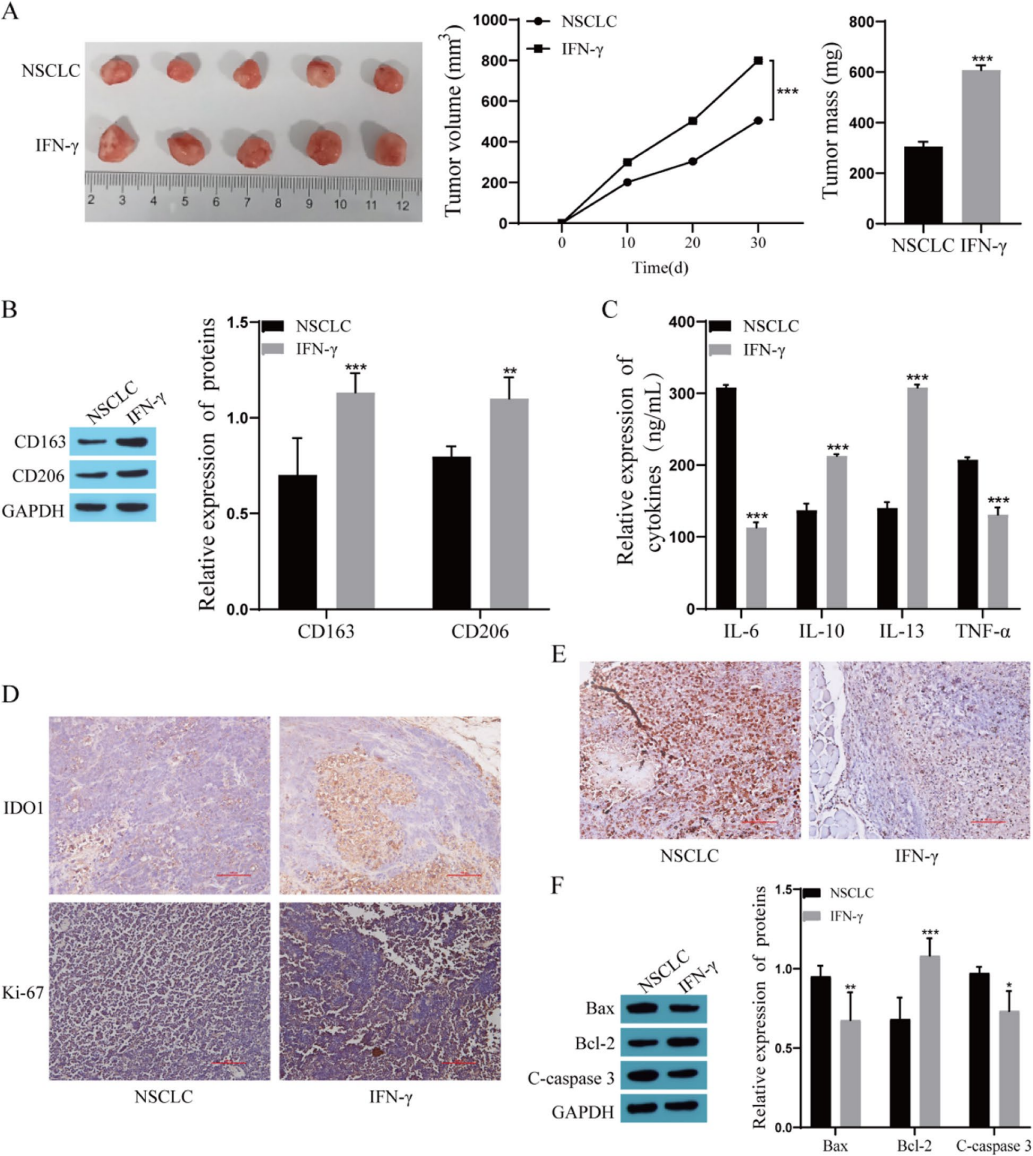
In vivo verification of IFN-γ promoting the M2 polarization of TAMs and the progression of stage IA NSCLC in mice. **A** Determination of tumor size, volume, and weight in mice; **B** CD163 and CD206 expression was detected by Western blot; **C**: IL-6, IL-10, IL-13 and TNF-α levels were measured by ELISA; **D** The expression of IDO1 and Ki-67 was detected by immunohistochemistry; **E** Apoptosis was evaluated by TUNEL staining; **F** The expression of Bax, Bcl-2 and C-caspase 3 was detected by Western blot. Compared with the NSCLC group, ^*^ *P* < 0.05, ^**^ *P* < 0.01, ^***^ *P* < 0.001

The NSCLC model was constructed by embedding small tumor pieces of stage IA NSCLC subcutaneously into the back of NOD SCID mice, and the mechanism by which IFN-γ promotes M2 polarization of TAMs and proliferation of stage IA NSCLC was verified in vivo. The tumor volume of the mice was recorded on Days 10, 20, and 30. After 30 days, the mice were sacrificed, and their tumor tissues were photographed and weighed. The results showed that the tumor volume and weight of mice in the IFN-γ-treated group were significantly larger than those in the NSCLC group (Fig. 6A). The Western blot results showed that the expression of CD163 and CD206, M2 macrophage markers, was significantly upregulated after IFN-γ treatment (Fig. 6B). The cytokine levels in the serum of the mice were measured by ELISA, and the results showed that the levels of IL-6 and TNF-α were significantly decreased, and the levels of IL-10 and IL-13 were significantly increased after IFN-γ treatment (Fig. 6C). The expression of IDO1 and Ki-67 in tumor tissues was detected by immunohistochemical staining, and the results showed that the expression of IDO1 and Ki-67 in the tumor tissues of mice in the IFN-γ group was significantly upregulated compared with that in the tumor tissues of mice in the NSCLC group (Fig. 6D). The TUNEL staining results showed that apoptosis was reduced after IFN-γ treatment (Fig. 6E). Further detection of the expression of apoptosis-related proteins showed that the expression of Bax and C-caspase 3 was significantly downregulated and the expression of Bcl-2 was significantly upregulated in the IFN-γ group compared with the NSCLC group (Fig. 6F).

## Discussion

Currently, the incidence of lung cancer is on the rise [26]. In the treatment of lung cancer, the focus of attention has shifted from the tumor itself to the TME. Tumor-associated macrophages are important cells involved in the formation of the tumor microenvironment [27]. A large number of studies have confirmed that TAMs promote the invasion and metastasis of lung cancer [28, 29]. In addition, the treatment of lung cancer is mainly dependent on the stage of lung cancer, but there are few reports about the relationship between different stages of lung cancer and TAMs. Previous studies explored the association between macrophages and tumor stage using NSCLC samples and found that there was no statistically significant correlation between tumor stage and TAMs [30]. However, other studies have also found heterogeneity in the infiltrated immune cells within the TME of stage IA NSCLC, specifically manifested as enriched M1 polarization of TAMs [31]. In addition, studies have reported that M2-TAMs can promote tumor growth and are associated with lower survival rates in lung cancer patients [32]. Stage IA NSCLC patients are prone to relapse and metastasis. Therefore, we further explored the potential relationship between TAM M2 polarization and the progression of stage IA NSCLC and found that IFN-γ can affect the progression of stage IA NSCLC by promoting TAM M2 polarization.

IFN-γ is a key modulator of cellular immunity, and in the TME, IFN-γ coordinates protumor and antitumor immunity [33]. The contradictory biological antitumor and protumor effects of IFN-γ have become the focus of research. The concentration of IFN-γ is an important factor in determining its function. For example, Mengjia Song et al. found that compared to a high dose of IFN-γ (100 ng/mL), a low dose of IFN-γ (0.1 ng/mL) can promote the stemness of NSCLC cells [34]. It has also been found that IFN-γ (10 ng/mL) can promote the immune evasion of lung cancer cells by upregulating the expression of PD-L1 [35]. In this study, the human lung adenocarcinoma cell line A549 was treated with different concentrations of IFN-γ (0, 50, 100, 150, 200, 250, 300 ng/mL). With increasing concentration, the proliferation activity of A549 cells gradually increased, but when the concentration reached 200 ng/mL, the proliferation activity of A549 cells did not continue to increase, and the proliferation activity of A549 cells decreased thereafter, which indicated that a low concentration of IFN-γ could promote the proliferation of lung adenocarcinoma cells, while a high concentration of IFN-γ inhibited proliferation. In addition, we treated A549 cells with 200 ng/mL IFN-γ and found that the percentage of apoptotic A549 cells was reduced, the expression of Bax and C-caspase 3 was downregulated, and the expression of Bcl-2 was upregulated, which promoted the proliferation, invasion and migration of A549 cells. It is suggested that IFN-γ at a concentration of 200 ng/mL can be used to explore the carcinogenic role of IFN-γ in stage 1A NSCLC.

Macrophages are the key cells of the innate immune system. They are the main components of the mononuclear phagocyte system and are found in almost all tissues. TAMs are composed of two major subtypes, M1 or M2 macrophages. The M1 type has antitumor properties, while the M2 type has protumor properties [36]. TAMs mainly have an M2-like phenotype, exhibiting an immunosuppressive state and promoting tumor progression. To date, it is believed that the M2-like phenotype is mainly induced by IL-4 or IL-13, while the M1-like phenotype is mainly induced by IFN-γ, so IFN-γ has antitumor properties [37, 38]. However, recent studies have found that IFN-γ signaling can promote the binding of immunosuppressive molecules to the surface of tumor cells and host myeloid cells to establish an immune microenvironment conducive to tumor progression, thus exhibiting protumor properties [19]. It can be speculated that IFN-γ may induce TAMs to polarize to the M2 phenotype and exert its tumor-promoting properties in stage IA NSCLC. TAMs make up a large proportion of TME immune cells, and data show a close relationship between the high infiltration of TAMs and poor prognosis in most types of tumors [39]. In this study, we established an organ model of the TME in patients with stage IA NSCLC to verify the effect of IFN-γ on the M2 polarization of TAMs in the stage IA NSCLC TME. When the TME organ model was treated with 200 ng/mL IFN-γ, the expression of the M2 polarization markers CD163 and CD206 was upregulated, indicating that 200 ng/mL IFN-γ induced the polarization of TAMs to the M2 phenotype. Tumor-specific CD8 + T cells are the core cells in the TME that exert antitumor effects, and CD4 + T cells can be used as an important prognostic factor for the immune response of the tumor microenvironment [40]. The downregulation of CD8 + and CD4 + in this study indicates a reduction in CD8 + T cell and CD4 + T cell numbers in the TME organ model. For apoptosis-related proteins, Western blot results showed an upregulation in the expression of Bcl-2 and a downregulation in the expression of Bax and C-caspase 3. Studies have shown that IL-6 and TNF-α are associated with the M1 polarization of TAMs, and IL-10 and IL-13 are associated with the M2 polarization of TAMs [41, 42]. Our ELISA results showed that after IFN-γ treatment, the levels of IL-6 and TNF-α were decreased, and the levels of IL-10 and IL-13 were increased. These results indicate that IFN-γ induces the polarization of TAMs to the M2 phenotype, establishes an immune microenvironment conducive to tumor progression.

In the tumor microenvironment, IDO1 is expressed by antigen-presenting cells such as macrophages, dendritic cells and tumor cells, and studies have shown that IFN-γ can induce IDO1 expression in macrophages at the transcriptional level [21, 22]. In addition, IDO1 expression in lung adenocarcinoma was found to indicate tumor progression [23]. This finding suggests that IDO1 may be an important regulatory protein causing tumor immunosuppression in the immune microenvironment of stage IA NSCLC. Our findings found that IDO1 expression was upregulated in the IFN-γ-treated TME organ model at 200 ng/mL. To further study the mechanism of TAM polarization to the M2 phenotype induced by IFN-γ, this study used PMA to induce THP-1 cells to differentiate into macrophages and explored the effect of IFN-γ on TAM polarization and its mechanism at the cellular level. IL-4 can induce TAMs to polarize to M2 [42], and after the successful induction by PMA, IL-4 stimulation upregulated expression of CD163 and CD206, decreased the levels of IL-6 and TNF-α, increased the levels of IL-10 and IL-13, and the phagocytic function of TAMs was suppressed. IFN-γ treatment further enhanced the effect of IL-4. Knockdown of IDO1 attenuated the effect of IFN-γ. The important immune cells in the tumor immune microenvironment include macrophages, T cells, and NK cells. TAMs can interact with other immune cell types in the tumor immune microenvironment, promote the activity of tumor-promoting immune cells, inhibit the function of tumor-killing immune cells, and promote the occurrence and development of tumors [43, 44]. In our study, IFN-γ-treated THP-1 cells were cocultured with A549 cells, T cells and NK cells, which enhanced the viability of A549 cells and reduced the viability of T cells and NK cells. In animal experiments, we also verified that IFN-γ can promote M2 polarization of TAMs and tumor growth.

**Fig. 7:**
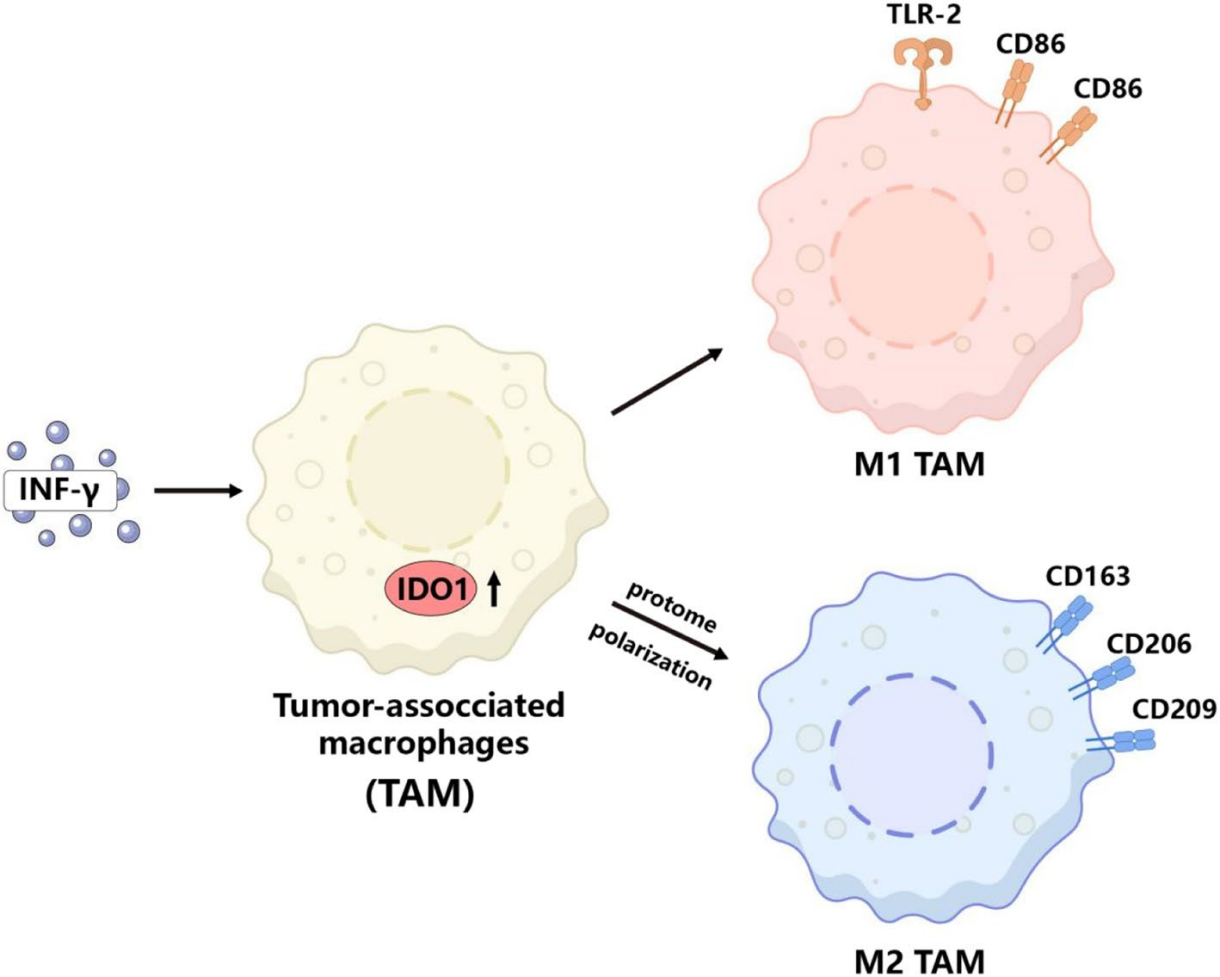
Mechanism diagram of IFN-γ promoting M2 polarization in tumor-associated macrophages via IDO1

In summary, our study found that IFN-γ promotes the polarization of TAMs to the M2 phenotype by upregulating the expression of IDO1 and suppressing the phagocytic function of TAMs on cancer cells, establishing an immune microenvironment conducive to tumor progression and thus promoting tumor progression (Fig. 7).

### Supplementary Information

**Additional file 1. **

## Data Availability

The datasets used and/or analyzed during the current study are available from the corresponding author upon reasonable request.
