# Supplementary material for: Effects of IFN-γ on the immunological microenvironment and TAM polarity in stage IA non-small cell lung cancer and its mechanisms
Source: BMC Pulm Med. 2024 Jan 22;24:46. doi: 10.1186/s12890-023-02809-6 (PMC10802021; doi:10.1186/s12890-023-02809-6)
Supplement: Supplementary file 1 — Additional file 1. [file 12890_2023_2809_MOESM1_ESM.pdf]

Figure 1F

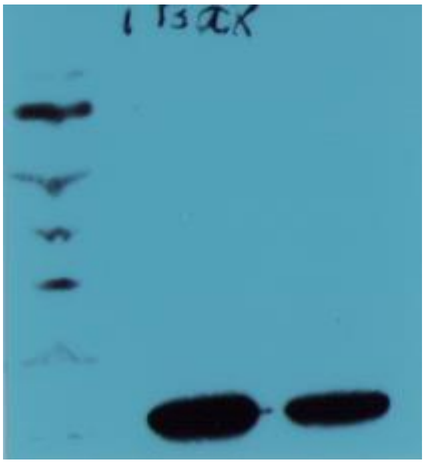

Bax

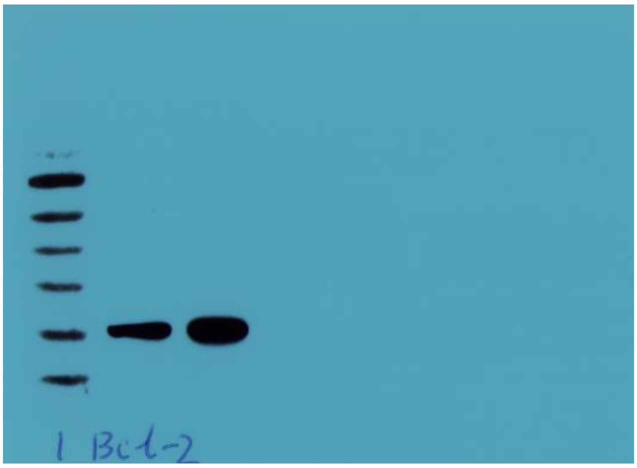

Bcl-2

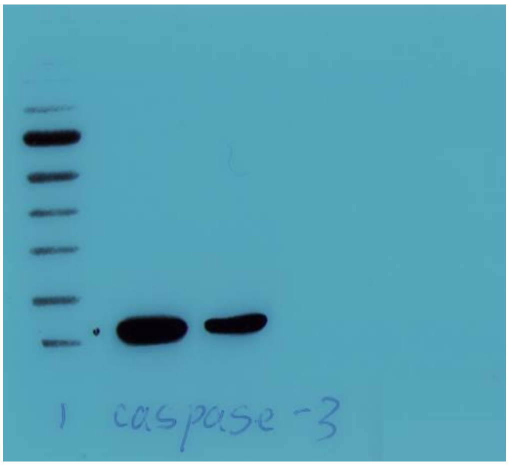

C-caspase3

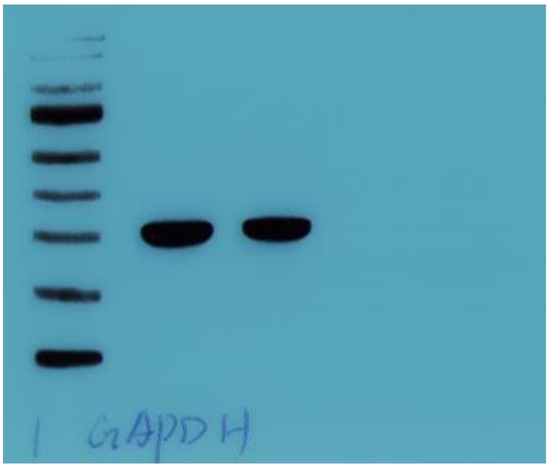

GAPDH

Figure 2A

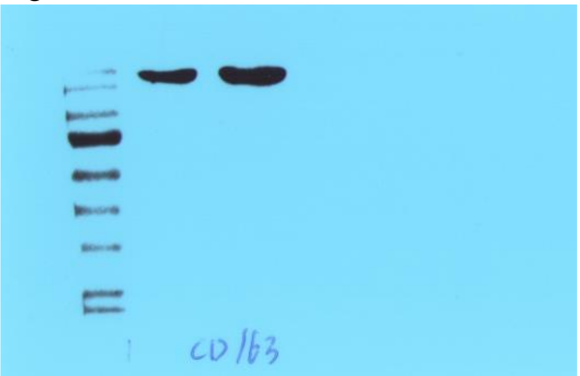

CD163

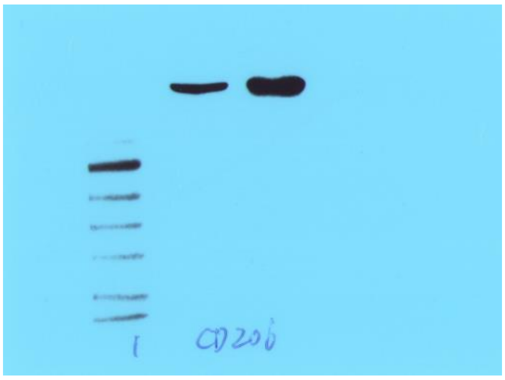

CD206

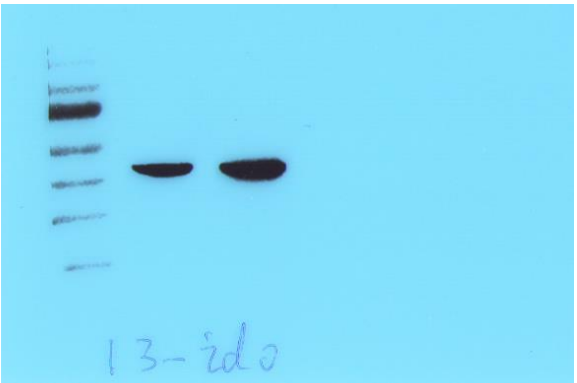

IDO1

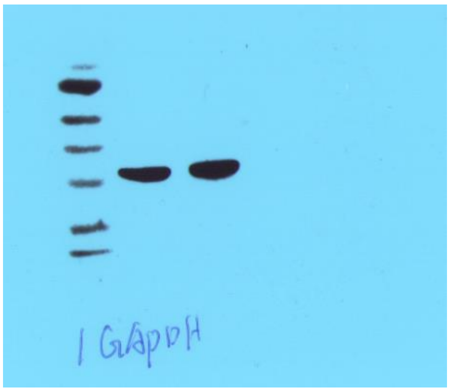

GAPDH

Figure 2B

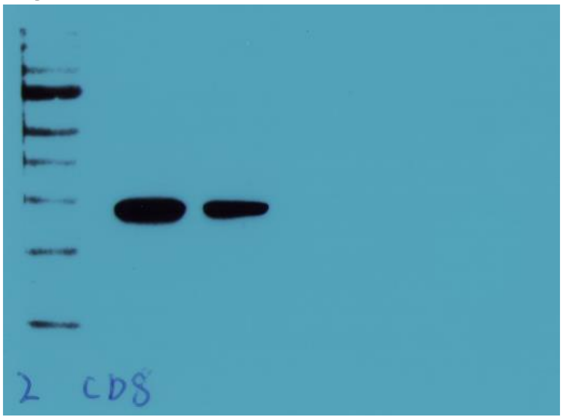

CD8

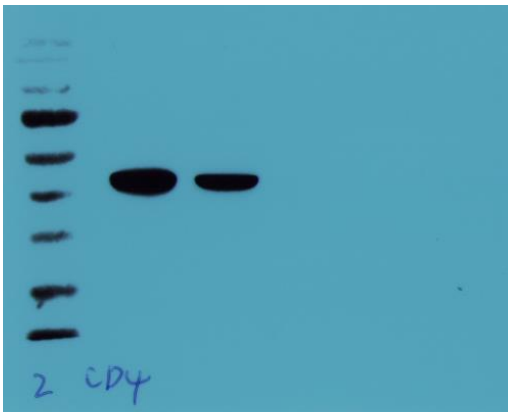

CD4

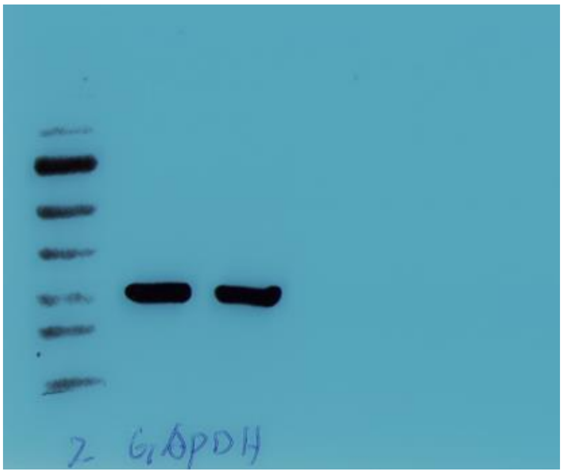

GAPDH

Figure 2C

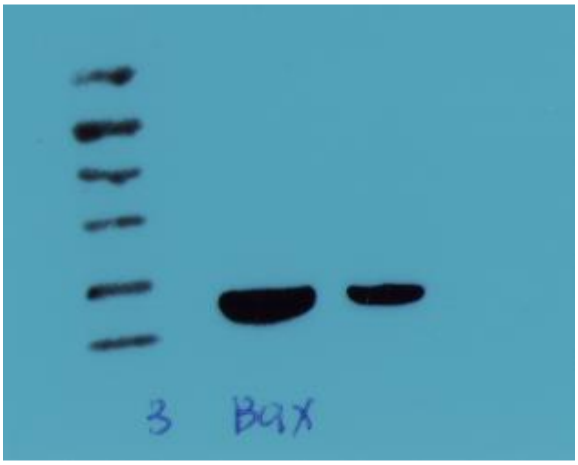

Bax

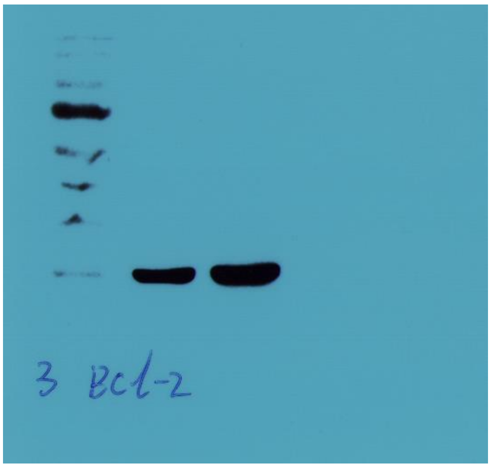

Bcl-2

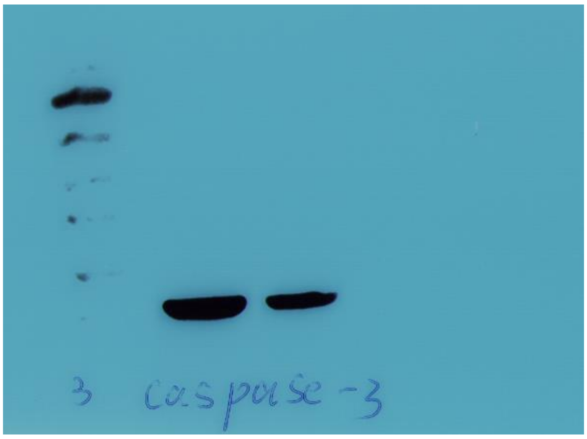

C-caspase3

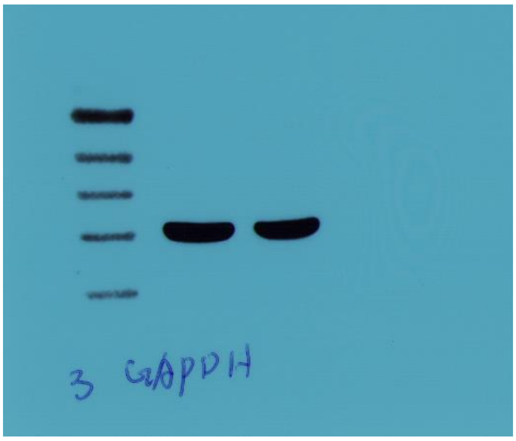

GAPDH

Figure 3B

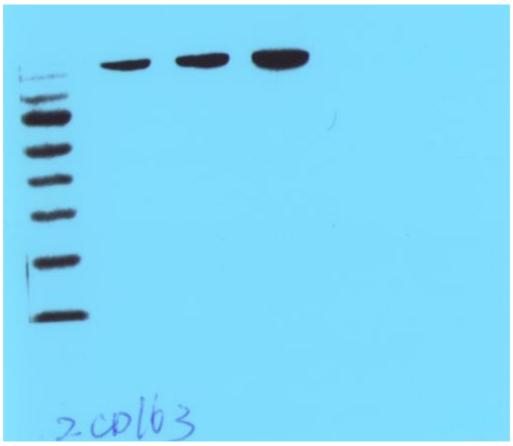

CD163

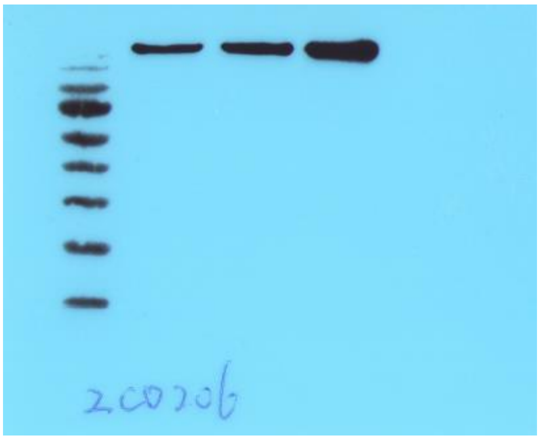

CD206

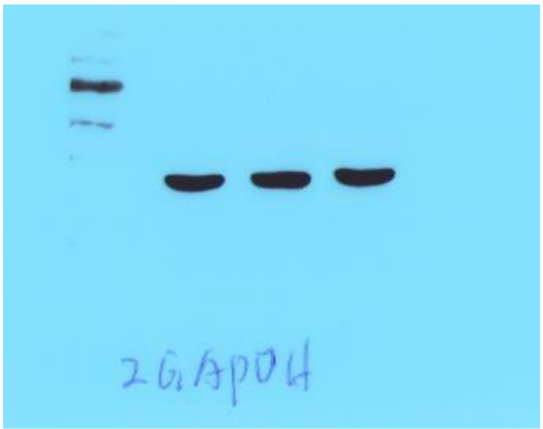

GAPDH

Figure 4A

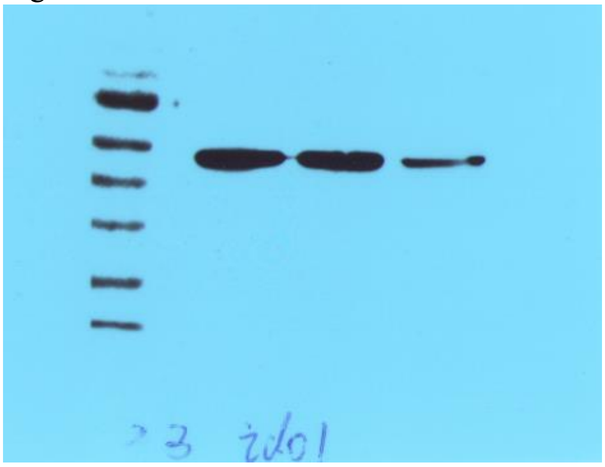

IDO1

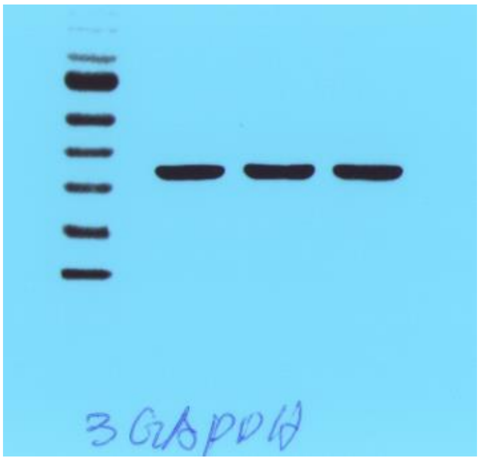

GAPDH

Figure 4B

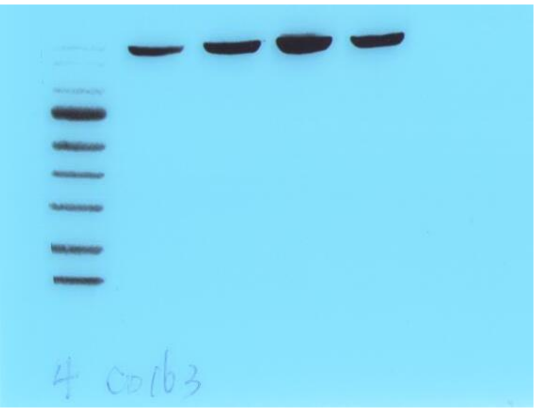

CD163

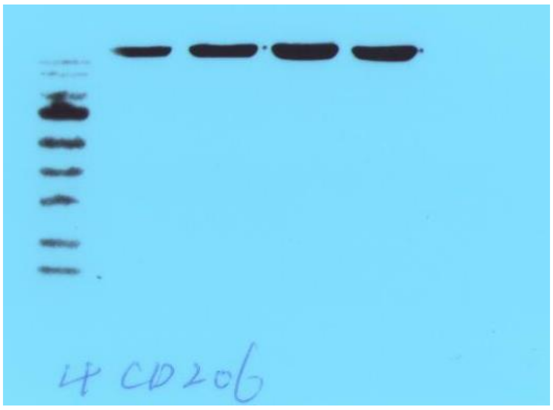

CD206

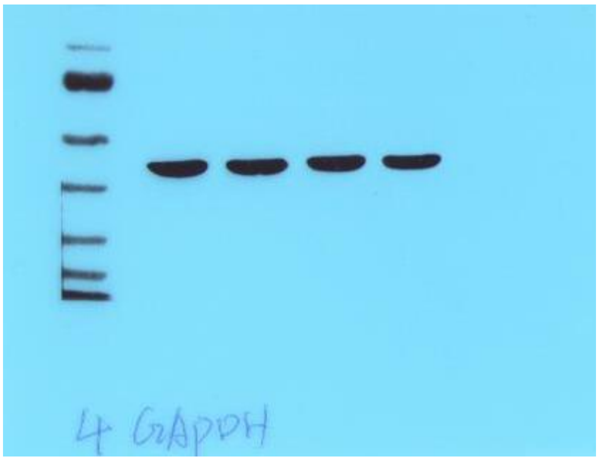

GAPDH

Figure 6B

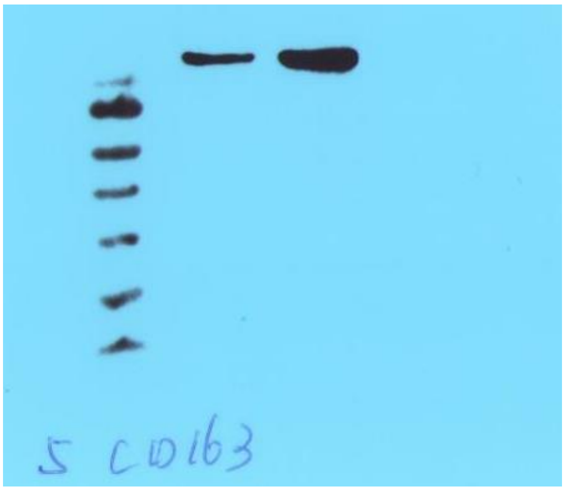

CD163

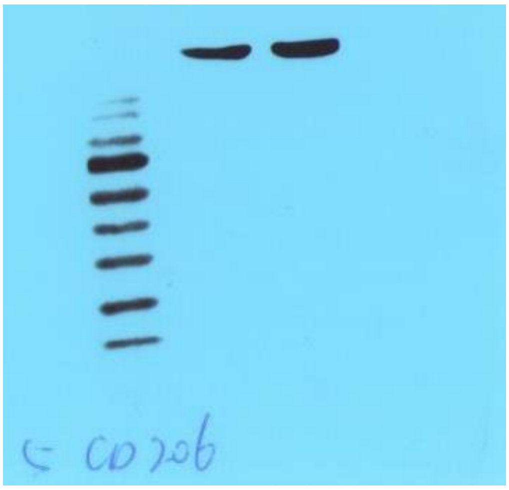

CD206

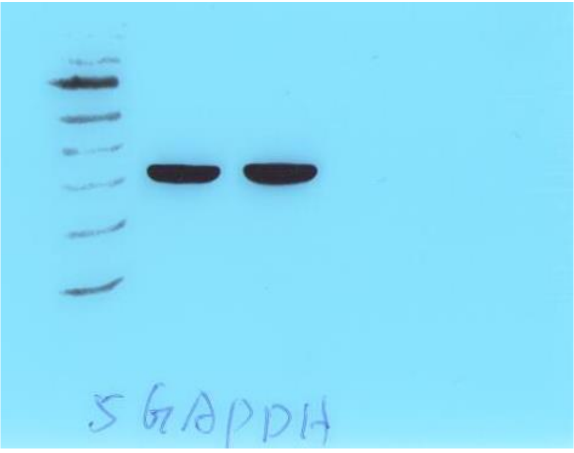

GAPDH

Figure 6F

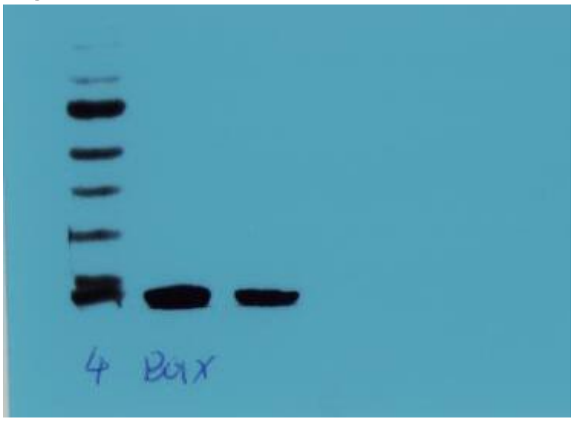

Bax

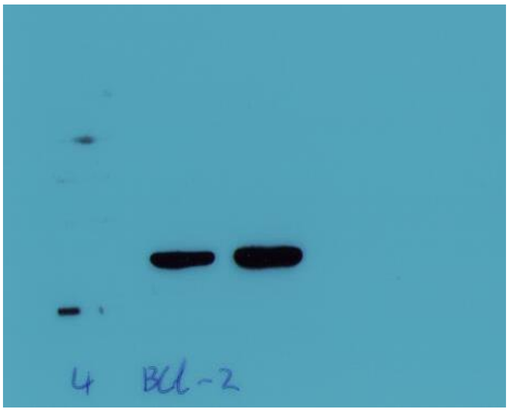

Bcl-2

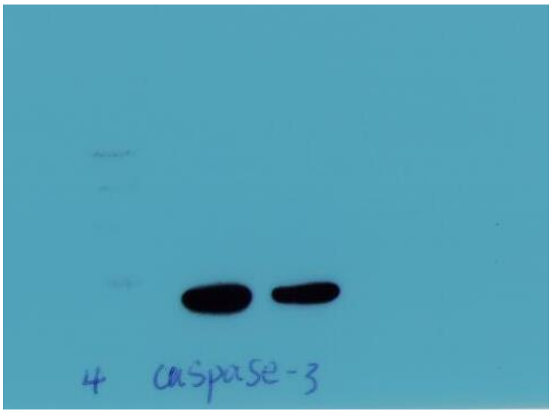

C-caspase3

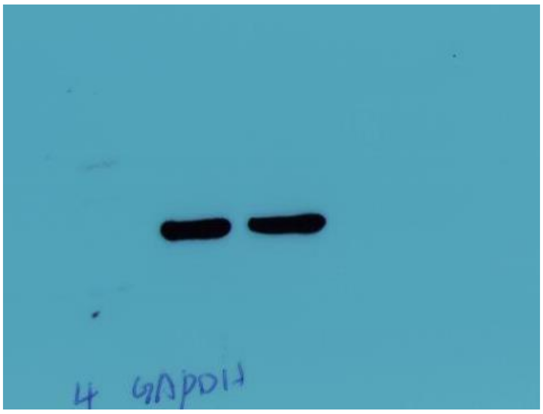

GAPDH
